# CD57 expression by T cells in the female genital tract of HIV-zx1 infected women

**DOI:** 10.1016/j.clim.2009.12.007

**Published:** 2010-04

**Authors:** Lenine J. Liebenberg, Adebayo Lawrence Adedeji, Darren P. Martin, Pam P. Gumbi, Lynette Denny, Jo-Ann S. Passmore

**Affiliations:** a Institute of Infectious Disease and Molecular Medicine, University of Cape Town, Observatory, Cape Town, South Africa; b Department of Biochemistry, Ladoke Akintola University of Technology, Nigeria; c Department of Obstetrics and Gynaecology, University of Cape Town, Observatory, Cape Town, South Africa; d National Health Laboratory Services, Cape Town, South Africa

**Keywords:** Cervical, Mucosal, CD8, Senescence, CD57, HIV

## Abstract

Despite an influx of T cells to the cervix during HIV infection, genital T cells are not associated with control of HIV shedding. CD57 expression by T cells has been associated with enhanced migratory potential and CD57+ T cells have been shown to accumulate in tissues during the late stages of HIV disease. We investigated the impact of HIV-infection and clinical status on the expression of CD57 by T cells from the female genital tract in 13 HIV-infected and 5 uninfected women. We found that cervical and blood-derived T cells expressed similar frequencies of CD57. The frequency of CD57 expression by cervical or blood T cells was not associated with clinical status (CD4 counts). No impairment in IFN-γ production by CD57+ T cells from the genital tract was observed. We conclude that increased T cell senescence does not appear to be a hallmark of genital mucosal HIV-1 infection.

## Introduction

Women infected with HIV-1 are significantly more susceptible to bacterial vaginosis and sexually transmitted infections than uninfected women [1–3] suggesting that local immunity in the genital tract is compromised during HIV infection. HIV-infected women have both significantly elevated concentrations of genital tract inflammatory cytokines and cellular recruitment than uninfected women [4]. Despite the generalized influx into the genital mucosa of HIV-specific cytotoxic T lymphocytes (CTLs) and other immune cells during HIV infection, HIV-responsive genital T cells are not associated with local control of HIV shedding [5,6].

In blood, progressive chronic HIV infection has been associated with terminal differentiation of T cells, increased expression of CD57 (a marker of senescence), decreased proliferative capacity and impaired cytolytic activity [7–9]. Effector memory T cells, the most terminally differentiated T cell subset found in the blood [7,10], are also the predominant subset of T cells found at the cervix of both HIV-infected and uninfected women [4]. CD57+ T cells have been shown to have enhanced migratory potential [10] and accumulate in lung mucosal tissue during the late stages of HIV disease [11]. We hypothesize that an impairment of mucosal T cell function associated with increased senescence (marked by CD57 expression) may contribute to the inability of T cells in the genital mucosa to control HIV locally. The aim of this study was to determine whether CD57 expression by T cells in the female genital tract was associated with genital mucosal HIV-1 infection.

## Methods

Thirteen HIV-1 subtype C infected and five uninfected women were recruited for this study. HIV-infected women had CD4 counts > 300 cells/µl. Women who were menstruating at the time of sampling, were post-menopausal, had undergone a hysterectomy, had visible or reported STI were excluded from the study. The study was approved by the University of Cape Town (South Africa) Research Ethics Committee and informed written consent was obtained from all volunteers before the study was initiated.

### Collection and processing of cervical specimens and blood

A cervical cytobrush was taken from women under speculum examination. Cervical mucosal mononuclear cells (MMCs) were collected as previously described [4,5] by inserting a Digene cervical sampler into the endocervical os, rotating 360° and placing the cytobrush in 3 ml transport media [RPMI1640 supplemented with 5 mM glutamine, fungazone, penicillin, streptomycin and 10 % fetal calf serum (FCS)]. Samples with visible blood contamination were discarded. Cells were processed within 4 h of collection by flushing the cytobrush ∼ 30 times with the same 3 ml transport media. The cell suspension was then transferred to a clean 15 ml tube and centrifuged at 2300 rpm (1000×*g*) for 10 min. The pelleted cells were resuspended in R10 prior to processing both for counting and phenotyping using an automated Guava cell counter (Guava technologies, Hayward, CA), and for functional analysis by flow cytometry (LSRII, Becton Dickinson (BD) Biosciences, San Jose, CA). Isolated mucosal T cells were quantified with the phenotypic marker, CD3-PE (T cells; Guava technologies) using a Guava Automated Cell counter (described in detail by Nkwanyana et al. [4]). Briefly, cells (25 μl/tube) were stained with pre-titrated anti-CD3-PE and incubated at 4 °C for 30 min. Cells were washed with 1 ml wash buffer (1% FCS PBS) and centrifuged at 1500 rpm (437×*g*) for 5 min. The supernatant was discarded and a volume of 200 µl Cell Fix (BD Biosciences) was added to each tube. Samples were acquired and analyzed using Cytosoft® software (Guava technologies). At least 2000 events were captured per analysis. Peripheral blood mononuclear cells (PBMCs) were isolated from ACD-anticoagulated whole blood using Ficoll-Hypaque density gradient centrifugation.

### Flow cytometry and intracellular cytokine staining

PBMCs (1 × 10^6^ cell/ml) or MMCs (∼ 0.15-1 × 10^6^ cell/ml) were stimulated with (i) HIV subtype C (Du422) Gag overlapping peptides (121 15-mer peptides overlapping by 10-amino acids; each peptide was used at a final concentration 1 μg/ml; peptides were kindly provided by the NIH AIDS Reagent Repository); (ii) PMA/ionomycin (each at a concentration of 10 μg/ml; Sigma-Aldrich; positive control); or (iii) untreated for 6 h at 37 °C and 5% CO_2_. Brefeldin A (10 μg/ml; Sigma, St. Louis, MO) was added after the first hour. The cells were washed in 10% FCS PBS containing 0.01% NaN_3_ (staining buffer) for 5 min at 300×*g* before staining with anti-CD3, CD57, CD4 and CD8 antibodies (Becton-Dickinson, San Jose, CA) for 30 min on ice. Cells were washed as before, were fixed and permeabilized (CytoFix/CytoPerm; BD), and washed with 0.1% Saponin (Fluka) in staining buffer. Cells were stained with anti-IFN-γ antibody (BD) for 1 h at 4 °C, were washed and then fixed with Cell Fix (BD). Fluorescence was measured using a FACSCalibur Flow Cytometer (BD Immunocytometry Systems [BDIS]) and FlowJo (Tree Star, Inc.) was used for analysis and compensation. From cytobrush samples, the median number of CD3+ events captured for flow cytometry was 1640 (range 100–10091).

For evaluation of surface expression of CD57, CD28, CD95 and CD38 expression by cervical and blood-derived T cells, an 8-colour panel including CD3, CD4, CD8, Vivid, CD57, CD28, CD95 and CD38 antibodies (Becton-Dickinson, San Jose, CA) was used. Cells were washed in 10% FCS PBS containing 0.01% NaN_3_ (staining buffer) for 5 min at 300×*g* before staining with surface antibodies (Becton-Dickinson, San Jose, CA) for 30 min on ice. Cells were washed as before, and then fixed with Cell Fix (BD). Fluorescence was measured using a LSRII Flow Cytometer (BD Immunocytometry Systems [BDIS]) and FlowJo (Tree Star, Inc.) was used for analysis and compensation. Fluorescence minus one was used to define gates.

### Measurement of viral load in cervical secretions

Viral load was determined in cervical secretions (derived from the cytobrush wash fraction) using Nuclisens Easyq HIV 1 Version 1.2. The detection limit of this assay was 50 copies/ml.

### Measurement of cytokine concentrations in genital secretions

Concentrations of inflammatory IL-1β, IL-6, and IL-8 and TNF-α in cervical supernatants were determined using Quantikine high sensitivity ELISA kits (R&D Systems Inc., Minneapolis, MN) according to manufacturer's instructions. The limit of detection of IL-1β, IL-6, IL-8 and TNF-α assays ranged between 0.6 and 1 pg/ml. Cytokine values below the assay's limit of detection were reported as zero.

### Statistical analysis

For parametric data, the paired Student's *t*-test was used for matched comparisons, unpaired Student's *t*-test was used for unmatched comparisons and Pearson's correlation test was used to test the association between variables using GraphPad Prism 5.0® (San Diego, CA). For non-parametric data, a Wilcoxon test was used for matched comparisons and the Spearman rank test was used to test associations. Chi-squared tests were used to compare binomial data. *P*-values ≤ 0.05 were considered significant.

## Results

Thirteen women chronically infected with HIV-1 were recruited into our study to investigate the frequency and functional ability of CD57+ T cells in the genital tract (Table 1). Five age-matched healthy uninfected women from the same community were recruited as controls. HIV-infected women had a median CD4 cell count of 394 cells/μl (IQR 324-558) and median plasma viral load of 8250 copies/μl (IQR 4425-40500).

Cervical T cells were isolated from both HIV-infected and uninfected women by cytobrush with a single cytobrush yielding a median of 92160 CD3+ T cells (IQR 84704-93900) from HIV- women and 71456 CD3+ cells (IQR 32160-113872) from HIV+ women (Table 1). Although the yield of CD3+ T cells did not differ significantly between groups, HIV-infected women had a significantly skewed CD4:CD8 ratio compared to uninfected women (0.3:1 in HIV+ compared to 1.8:1 in HIV−; *p* = 0.0002; unpaired *t*-test) indicating that HIV+ women in general had fewer cervical CD4+ T cells than HIV− women (Table 1).

Of the HIV-infected women studied, 7/13 (53.8%) had detectable HIV RNA in their cervical secretions indicating that they were shedding virus (Table 1). Of the 7 women found to be shedding HIV, their median genital tract HIV load was 350 RNA copies/ml of cervical secretion (IQR 280-2450).

### Comparison of CD57 expression by CD4 and CD8 T cells at the cervix and in blood

**Figure 1:**
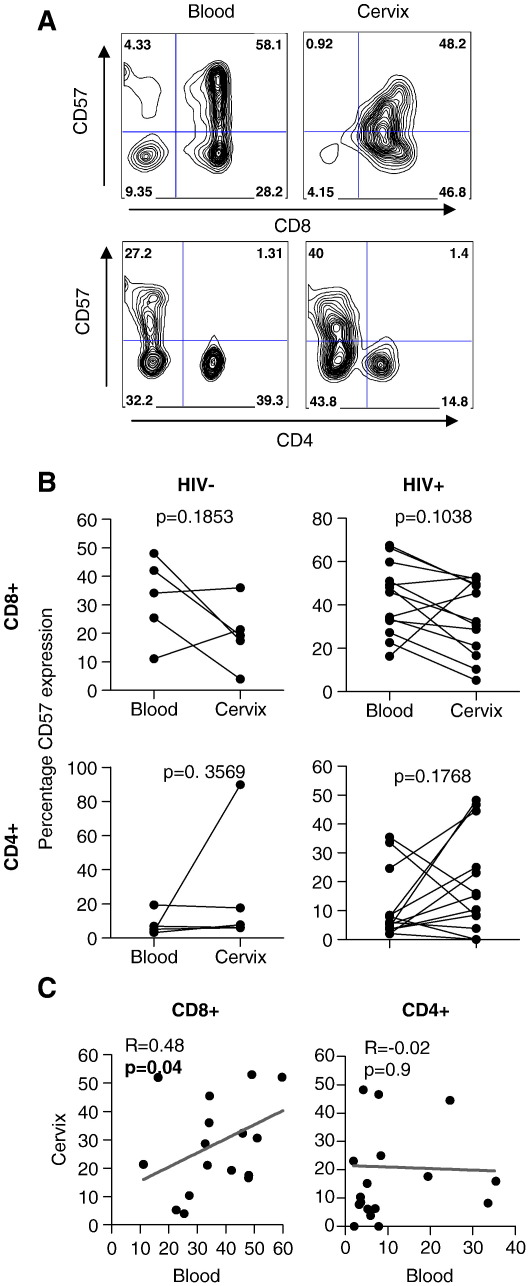
Expression of CD57 by CD8+ and CD4+ T cells derived from the cervix and blood of HIV+ and HIV− women. (A) Representative plots showing the frequency of CD57 expression by blood (left panels) and cervical (right panel) CD8+CD3+ (top panels) and CD4+CD3+ (lower panels) T cells. (B) We compared the frequency of CD57+ expression by CD8+CD3+ (upper panel) and CD4+CD3+ (lower panel) T cells in blood and cervical T cells from HIV− (*n* = 5) and HIV+ (*n* = 13) women. Each dot represents an individual woman's CD57 frequency and lines joining dots indicate matched blood and cervical frequencies in individual woman. Paired Student's *t*-test was used to compare CD57 expression frequencies in blood and at the cervix. An unpaired Student's *t*-test was used to compare CD57 expression frequencies in HIV+ and HIV− women. (C) Association between the frequency of CD57 expression by cervical and blood-derived CD8+CD3+ (left panel) and CD4+CD3+ (right panel) T cells. Regression lines and Pearson *R*-values are shown for correlations. *p*-values ≤ 0.05 were considered significant.

The frequency of CD57 expression on CD8 and CD4 T cells present at the cervix of HIV-infected and uninfected women was compared to frequencies detected in peripheral blood (Fig. 1). A representative plot showing CD57 expression by CD8 and CD4 T cells from blood and cervix is shown in Figure 1A. HIV-infected and uninfected women had similar frequencies of CD8+ T cells expressing CD57 irrespective of compartment (Fig. 1B). CD4+ T cells expressed significantly less CD57 both in blood and at the cervix than CD8+ T cells (*p* < 0.0001 in blood and *p* = 0.002 at the cervix) and the frequency of CD57 expression by CD4+ T cells was similar irrespective of HIV status (Fig. 1B). In addition, we observed no difference in the frequency of CD57 expression by T cells isolated from the cervix and blood irrespective of HIV status (Fig. 1B). We found a significant correlation between the frequency of CD57 expressing CD8+ T cells in blood and at the cervix indicating a relatedness between the compartments (*R* = 0.48; *p* = 0.04) although this was not the case for CD4+ cells (Fig. 1C).

### Relationship between CD57 expression frequencies and clinical status (CD4 counts)

We compared the frequency of CD57 expression by cervical and blood T cells with markers of HIV disease status (blood CD4 counts). HIV-infected women with > 500 CD4+ T cells/ml had similar frequencies of CD57 expression by CD8+ and CD4+ T cells in blood as women with < 500 CD4 cells/ml (Table 2). In contrast, HIV-infected women with better CD4+ counts had 1.6-fold lower frequencies of CD57+ CD8 T cells at the cervix than women with CD4 counts < 500 cells/μl (although this was not significant; *p* = 0.6). We have previously shown that the CD4:CD8 ratio of genital tract-derived T cells correlate significantly with blood CD4 counts [4] and confirm this in the present study (*p* = 0.04; Pearson *R* = 0.48). We observed no correlation, however, between cervical CD57 expression and the cervical CD4:CD8 ratio (data not shown).

### Association between CD57 expression and other markers of senescence or activation

**Figure 2:**
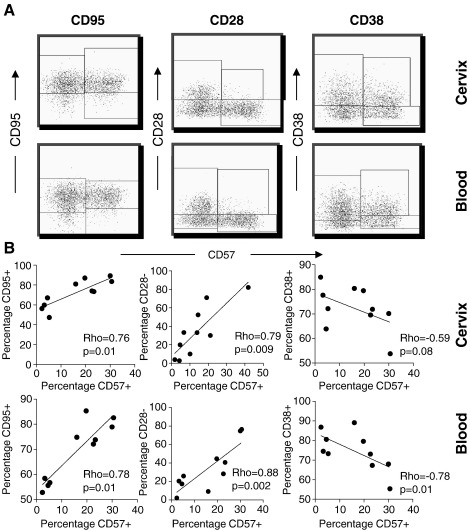
Comparison of CD95, CD28 and CD38 expression by CD57+ T cells in the female genital tract and blood. (A) Representative plots showing the frequency of CD57 expression by cervical (top panels) and blood (right panel) CD8+CD3+ T cells co-expressing CD95 (left panels), CD28 (middle panels) or CD38 (right panels). (B) Association between the frequency of CD57 expression by cervical (top panels) and blood-derived (bottom panels) T cells and CD95 expression (left panels), lack of CD28 expression (CD28−; middle panels) and CD38 expression (right panels). Regression lines and Spearman Rho-values are shown for correlations. *p*-values ≤ 0.05 were considered significant.

We evaluated expression of other markers of T cell activation/senescence (CD28, CD95 and CD38) by CD57+ T cells derived from the cervix and blood (Fig. 2). Figure 2A shows representative plots of CD8+ T cells from the cervix (upper panels) and blood (lower panels) co-expressing CD57 with CD95, CD28 or CD38 in HIV-infected women. We observed a significant positive correlation between surface expression of CD57 and CD95 by both blood and cervical T cells (Fig. 2B; Rho = 0.78, *p* = 0.01 for blood and Rho = 0.76, *p* = 0.01 for cervix). Similarly, we found a significant association between expression of CD57 and lack of expression of CD28 (CD28−; Rho = 0.88, *p* = 0.002 for blood and Rho = 0.79, *p* = 0.009 for the cervix) indicating that expression of CD28 and CD57 was largely mutually exclusive. Finally, the marker of activation CD38 was predominantly expressed by CD57- T cells and these markers were significantly negatively correlated (Rho = −0.78, *p* = 0.01 for blood and Rho = −0.59, *p* = 0.08 for the cervix). These data indicate that, irrespective of compartment, the majority of CD57+ T cells (65-99%) co-expressed the apoptotic marker CD95 (FasL). T cells expressing the co-stimulatory molecule CD28, associated with less terminally-differentiated T cell subsets, were not likely to co-express CD57 in both the cervix and blood.

### Comparison of CD57+ versus CD57- function by cervical and blood T cells in response to stimulation with PMA/ionomycin

**Figure 3:**
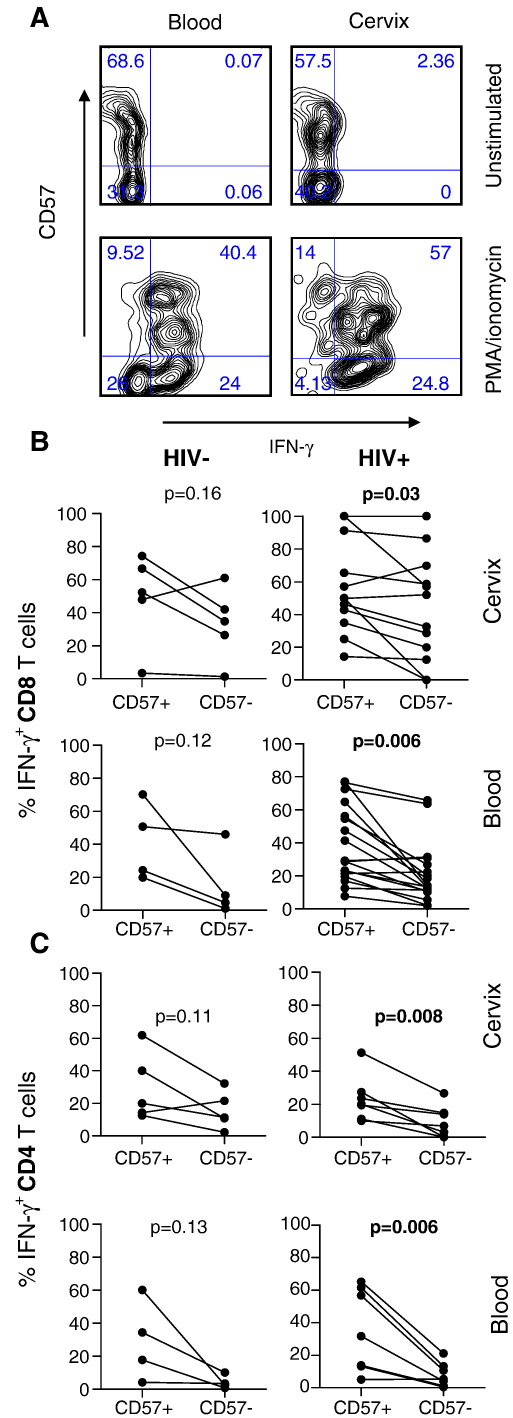
Comparison of ex vivo IFN-γ production by CD57+ and CD57− T cells derived from the blood and genital mucosa of HIV− and HIV+ women in response to polyclonal stimulation (PMA/ionomycin). (A) Representative plots showing the frequency of IFN-γ production by CD57+ or CD57− CD8^+^ T cells in the blood (left panels) and cervix (right panels) following PMA/ionomycin stimulation. (B) Net percentages of IFN-γ producing CD57+CD8+ and CD57−CD8+ T cells (percentage of stimulated IFN-γ producing cells minus the percentage of background percentage of unstimulated cells producing IFN-γ) after ex vivo stimulation with PMA/ionomycin were compared in cervical mucosa (upper panel) and blood (lower panel) in HIV− (left panel) and HIV+ (right panel) women. (C) Net percentages of IFN-γ producing CD57+CD4+ and CD57−CD4+ T cells in cervical mucosa (upper panel) and blood (lower panel in uninfected (left panel) in HIV− and HIV+ (right panel) women. Each dot represents an individual's net percentage of CD57+CD8+ or CD57−CD8+ T cells producing IFN-γ at the cervix and in blood. *p*-values ≤ 0.05 were considered significant. Paired Student's *t*-test was used to compare IFN-γ frequencies by CD57+ versus CD57− T cells per individual.

To assess the relationship between IFN-γ production and CD57 expression in the blood and cervix, CD57^+^ and CD57^−^ blood- and cervix-derived T cells from chronically HIV-infected women were assessed by flow cytometry for IFN-γ responses following stimulation with PMA/ionomycin (Fig. 3). Figure 3A shows representative plots of IFN-γ production by CD57+ and CD57− CD8+ T cells from the cervix and blood. In blood and at the cervix of HIV-infected women, significantly higher frequencies of CD57+CD8+ T cells produced IFN-γ than CD57-CD8+ T cells (*p* = 0.006 for blood and *p* = 0.03 for cervical samples; Fig. 3B). This trend was also observed in uninfected women although this was not significant.

Similarly, in the CD4+ T cell population (Fig. 3C), significantly higher frequencies of CD57+ cells produced IFN-γ than CD57- cells (*p* = 0.006 for HIV+ blood and *p* = 0.008 for HIV+ cervical samples). There was also a trend towards higher frequencies of IFN-γ production by CD57+ cells than CD57− cells in the blood and cervix from uninfected women but this was not significant.

### Comparison of CD57+ versus CD57− function by cervical and blood T cells in response to stimulation with HIV Gag peptides

**Figure 4:**
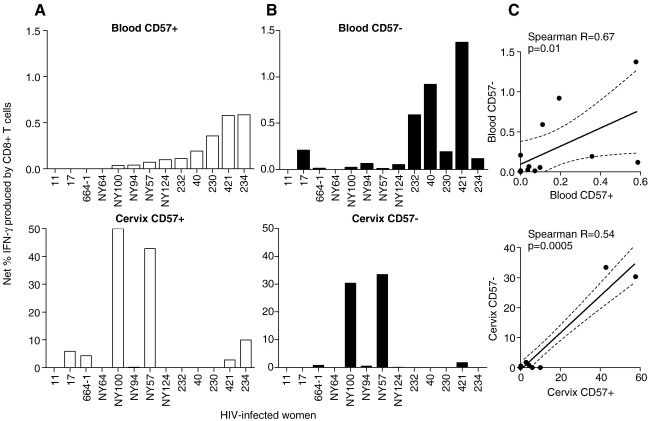
Comparison of ex vivo IFN-γ production by CD57+ and CD57− T cells derived from blood and the genital mucosa of HIV+ and HIV− women in response to stimulation with HIV Gag peptides (Du422 Subtype C). (A) Net percentages of CD57+CD8+ T cells in HIV+ women (*n* = 18) producing IFN-γ (percentage stimulated IFN-γ producing cells minus the percentage of unstimulated cells producing IFN-γ) after ex vivo stimulation with HIV Gag peptides were compared in blood (upper panel) and at the cervical mucosa (lower panel). (B) Net percentage of CD57−CD8+ T cells from HIV+ women producing IFN-γ in blood (upper panel) and at the cervix (lower panel). Each bar represents an individual's net percentage IFN-γ producing CD57+CD8+ or CD57−CD8+ T cells at the cervix and in blood. (C) Association between the net percentages of CD57+CD8+ and CD57−CD8+ cells producing IFN-γ in response to Gag in the blood (upper panel) and at the cervix (lower panel). Each dot represents an individual woman's net IFN-γ producing CD57+ and CD57− T cell frequencies in blood and at the cervix. Regression lines and Spearman Rho values are shown for correlations. *p*-values ≤ 0.05 were considered significant. Wilcoxon matched pairs tests were used to compare frequencies of IFN-γ producing CD57+ and CD57− T cells within and between compartments.

CD57^+^ and CD57^−^ blood- and cervix-derived T cells from chronically HIV-infected women were assessed for IFN-γ responses following stimulation with HIV-1 subtype C Gag peptides (Fig. 4). While 9/13 (69.2%) of the HIV-infected women studied had detectable HIV-specific responses in blood, only 6/13 (46.2%) had matching responses detected at the cervix (*p* = 0.2, Chi-squared test). The frequency of IFN-γ positive cells in these 6 women who did have cervical T cell responses was significantly higher than the frequency of responses detected in matching blood samples (*p* = 0.03). In women with HIV Gag-specific responses in both blood (upper panels in Fig. 4) and at the cervix (lower panels in Fig. 4), IFN-γ production was detected in both CD57+ CD8+ (Fig. 4A) and CD57− CD8+ T cells (Fig. 4B) indicating that both subsets were contributing to Gag-specific immunity. In the genital tract, however, we found that the frequency of Gag-specific IFN-γ responses was significantly higher in CD57+ cells than CD57− cells (*p* = 0.04). We observed a significant positive association between the net frequencies of IFN-γ producing CD57+ and CD57− cells within each compartment (Fig. 4C; Spearman *R* = 0.67 and *p* = 0.01 in blood; *R* = 0.54 and *p* = 0.0005 at the cervix).

### Association between genital tract inflammation, HIV shedding and CD57 expression

We compared the concentrations of inflammatory cytokines IL-6, IL-8, IL-1β and TNF-α in genital secretions from women infected with HIV and those who were not (Table 3). HIV-infected women had significantly elevated genital tract concentrations of IL-6, IL-8 and IL-1β than uninfected women. However, we observed no association between genital inflammatory cytokine concentrations and the frequency of CD57 expression by cervical T cells (Table 3).

HIV RNA was detected in the cervical secretions from 7/13 (53.8%) of HIV-infected women studied indicating that they were shedding virus (Table 1). We found that HIV-infected women shedding HIV had similar concentrations of inflammatory cytokines in genital secretions compared to women who were not shedding (Table 4). Since we found a range of CD57 expression frequencies at the cervix of HIV-infected women (ranging from 14.2% to 100%), we investigated whether the extent of T cell exhaustion in the female genital tract was associated with HIV genital shedding (Table 4). We found no significant difference in the frequency of CD57 expression by cervical CD8+ T cells from HIV shedders versus non-shedders (median of 21.1% CD57+ for shedders compared to 33.3% for non-shedders; *p* = 1.0). In contrast, cervical CD4+ T cells from women shedding HIV had ∼ 2.5-fold higher frequencies of CD57 expression than women not shedding although this was not significant (median of 20.1% CD57+ for shedders compared to 8.4% for non-shedders; *p* = 0.2).

## Discussion

In the genital tract, we and others have previously shown that the presence of HIV-specific T cells was not associated with local control of HIV shedding [5,6]. CD57 has been identified as a marker of replicative senescence and impaired cytolytic function by T cells during chronic HIV infection [7,12–14], and T cells expressing CD57 have also been shown to accumulate in lung mucosal tissue during the late stages of disease [11]. Since genital tract infiltrating T cells have been shown to be predominantly differentiated effector memory phenotype [4] and since there have been no published studies of CD57 expression at the female genital mucosa, we hypothesized that CD57 expression by cervical T cells may be associated with impaired T cell function resulting in inability to control local HIV replication.

We first investigated whether CD57+ T cells were more prevalent in the cervices of HIV-infected women than they were in uninfected women and then whether CD57 expression frequencies were associated with markers of disease progression or decreased T cell function. In blood, we found no significant difference in the frequencies of CD57+ T cells in HIV infected and uninfected women. While some studies have reported higher frequencies of blood CD57 expression associated with HIV infection [10,15–17], others did not find any difference [11]. At the cervix, similar frequencies of CD57+ T cells were observed at the cervices of both HIV− and HIV+ women. Irrespective of the compartment from which T cells were isolated, we found that the frequency of CD8+ cells expressing CD57 was significantly higher than the frequency of CD4+ T cells expressing this marker. Further, we showed that the frequency of CD57 expression by cervix- and blood-derived CD8+ T cells was significantly correlated.

Wood et al. [11] found that HIV-infected women with CD4 counts < 500 cell/μl had significantly elevated frequencies of CD57+ T cells at the lung mucosa compared to uninfected women and HIV+ women with higher CD4 counts (> 500 cells/μl). These earlier studies conducted in the lung were done using cells isolated from bronchoalveolar lavage [11] which is comparable to cervical cytobrushing in terms of the depth of sample collection from mucosal surfaces. We did not observe any significant association between the frequency of CD57 expression by cervical T cells and clinical status (CD4 counts). The 13 HIV+ women included in the current study had been infected with HIV for a median of 7 years (IQR 5.6–7.5 years) with the majority having CD4 counts < 500 cells/μl (64%). It is therefore possible that the differences in CD57 expression at the lung compared to blood [11] may have been masked in our study by inclusion of women in more advanced HIV disease. It would be beneficial in the future to focus analysis on HIV-infected women during early infection where compartment-specific differences may be more likely. It would be beneficial in future to focus analysis on HIV-infected women during early infection where compartment-specific differences may be more likely. In addition, ∼ half of the HIV+ women studied by Wood et al. [11] were taking anti-retroviral therapy while none of the 13 HIV+ included in this study were. Immune reconstitution associated with ART may be associated with reduced immune exhausting (CD57 expression) and may contribute to the discrepancies observed between our study and the findings reported by Wood et al. [11].

We found that significantly higher frequencies of CD57+ T cells in the female genital tract produced IFN-γ than CD57- T cells, indicating that CD57 expression was not associated with a decreased capacity to produce IFN-γ. The majority of cervical T cells derived through cytobrushing are generally effector memory cells (53% of CD8+CD3+ T cells at the cervix are CD45RA−CD27−CCR7− effector memory cells [4]) and effector memory T cells are the most terminally differentiated T cell subset [7,10]. It was therefore surprising that despite CD57 expression being a marker of terminal differentiation [7,12–14], the frequency of cervical T cells expressing CD57 is similar to and significantly correlated with that observed for cells in blood. It is noteworthy that cervical cells analyzed in this study, derived from outer epithelial layers of the genital tract by gentle cytobrushing, may not reflect the frequency or number of CD57 expressing cells in genital tissue (from cells derived by tissue biopsy).

Terminally differentiated (CD57+) T cells are preferentially recruited to the lung mucosa during the course of HIV-infection [11,16]. Relative to CD57- T cells, CD57+ T cells in blood are known to have increased expression of the chemokine receptor CX3CR1 and reduced expression frequencies of CCR7 and CD62 ligand [10]. Previous indications of increased CD8+ CD57+ T cell frequencies in lung tissues are consistent with CD57 expression predisposing CD8+ T cells to migrate to tissues rather than to lymphoid organs [19].

We found that CD57+ T cells derived from the cervix and blood were largely CD95+ indicating their propensity to undergo apoptosis. We observed similar frequencies of CD95+ T cells in blood and at the cervix (data not shown) suggesting the cells in the genital tract were not more prone to CD95-associated apoptosis than cells from blood. Previous studies have reported that ∼ 99% of senescent T cells in blood do not express CD28 and that CD28 expression is associated with naïve or less differentiated T cells [20]. In support of this, we found a reciprocal relationship between expression of CD57 and CD28 by T cells from the cervix and blood indicating that expression of these markers was mutually exclusive.

Since CD57 expression is enhanced by chronic antigenic stimulation in blood [10,15,17,18], we investigated HIV-specific IFN-γ responses by CD57+ T cells at the genital mucosa. Although we found no significant differences in the ability of CD8+CD57+ and CD8+CD57− T cells to secrete IFN-γ following PMA stimulation, HIV Gag-specific responses were more readily detected at the cervix for CD8+CD57+ T cells than they were for CD8+CD57− T cells. The frequencies of HIV Gag-specific CD8+CD57+ and CD8+CD57− T cells at the cervix are significantly higher than those in the blood during chronic HIV infections. This raises questions about both the effects of compartment-specific antigen loads on HIV-specific T cell responses, and the migratory capacity and patterns of HIV-specific CD8+CD57+ T cells. Although our study has measured the ability of T cells to secrete IFN-γ alone as a surrogate for T cell functional ability, the functional competence of T cells is better defined by broader capacity to secretion of additional cytokines and release perforin (polyfunctionality), direct cytolytic activity against target cells and, most importantly, the direct ability to proliferate [21].

This is the first study to investigate CD57 expression of cervical T cells ex vivo and to investigate the impact of CD57 expression on HIV-specific and mitogen-induced cytokine function at the cervix. The ability to mount an effective mucosal cellular immune response to an HIV vaccine is one of the fundamental prerequisites to providing prophylactic protection against infection. With increased susceptibility to opportunistic genital tract infections during HIV infection, enhanced cellular recruitment to the cervix, and the association between impaired proliferative capacity and lytic function of CD57+ T cells, concerns have been raised about the efficacy of genital tract T cells to control HIV infection. Since the frequency of CD57 expression by T cells from the genital tract largely mirrors that of the blood in chronic HIV infection, we found no obvious evidence of an association between CD57 expression and impaired cervical T cell function. We conclude that increased T cell senescence in the female genital tract does not appear to be a hallmark of genital mucosal responsiveness to HIV-1 infection.

## Figures and Tables

**Table 1:** Clinical details of the women included in the study.

Characteristic	HIV−	HIV+
*N*	5	13
Age	36 (34–48)	34 (29–37)
Duration of infection [years; median (IQR)]	na	7.0 (5.6–7.5)
CD4 count [cell/μl; median (IQR)]	na	394 (324–558)
Plasma HIV load [copies/μl; median (IQR)]	na	8250 (4425–40,500)
Number of women shedding HIV in the genital tract [*N*/Total (%)]	na	7/13 (53.8)
Genital tract viral load [copies/μl; median (IQR)]	na	350 (280–2450)
Macroscopic findings [*N*/Total (%)]⁎⁎	0/5 (0)	1/13 (7.7)
Cervical CD3 yield	92,160 (84,704–93,900)	71,456 (32,160–113,872)
Cervical CD4:CD8 ratio	1.8 (1.1–2.2)	0.3 (0.2–0.4)⁎

⁎ *p* = 0.0002.

⁎⁎ Macroscopic findings include vaginal discharge, candidiasis, genital ulcers, genital warts or any macroscopically visible findings of note under speculum examination.

**Table 2:** Impact of HIV clinical status (CD4 counts) on CD57 expression in blood and at the cervix.

Subset	Compartment	Percentage CD57 expression [median (IQR)]		*p*-value
		< 500 CD4 cells/μl (*n* = 9)	> 500 CD4 cells/μl (*n* = 4)	
CD8	Blood Cervix	45.9 (24.9–63.1) 48.9 (15.7–52.1)	41.2 (33.2–50.3) 29.7 (19.7–41.7)	0.9 0.6
CD4	Blood Cervix	5.9 (3.9–16.5) 8.5 (1.9–34.8)	5.8 (2.4–28.6) 19.5 (11.8–40.8)	0.8 0.5

**Table 3:** Association between genital tract inflammation and CD57 expression in the female genital tract.

Cytokines	HIV-infected median (IQR)	Uninfected median (IQR)	*p*-value	Correlation⁎ with cervical CD57 expression frequencies	
				*R*	*p*-value
IL-6 (pg/ml)	89.9 (49.4–183.8)	30.4 (5.9–57.4)	0.02	0.34	0.08
IL-8 (pg/ml)	80.7 (23.8–230.3)	25.4 (13.3–28.6)	0.04	0.24	0.27
IL-1β (pg/ml)	68.9 (11.2–172.3)	1.9 (1.3–11.1)	0.004	0.18	0.37
TNF-α (pg/ml)	0.0 (0.0–4.2)	0.0 (0.0–0.01)	0.4	0.27	0.18

⁎ Pearson correlation.

**Table 4:** Association between HIV shedding, inflammatory cytokine concentrations and CD57 expression in the female genital tract.

	HIV shedders⁎ median (IQR)	Non-shedders median (IQR)	*p*-value
IL-6 (pg/ml)	147.0 (23.4–183.9)	97.3 (40.4–195.7)	0.9
IL-8 (pg/ml)	80.2 (35.4–301.9)	81.3 (13.9–206.4)	0.5
IL-1β (pg/ml)	81.5 (34.4–150.9)	99.9 (7.7–171.0)	0.8
TNF-α (pg/ml)	0.02 (0–29.8)	0 (0–0)	0.2
% CD8+CD57+	21.1 (10.3–53.0)	33.3 (40.3–48.9)	1.0
% CD4+CD57+	20.1 (7.4–60.0)	8.4 (5.0–23.1)	0.2

⁎ HIV shedders were defined as any HIV-infected women with HIV RNA copies > 50 copies/ml of cervical supernatant.
